# Increased Body Exposure to New Anti-Trypanosomal Through Nanoencapsulation

**DOI:** 10.1038/s41598-017-08469-x

**Published:** 2017-08-16

**Authors:** Renata Tupinambá Branquinho, Gwenaelle Pound-Lana, Matheus Marques Milagre, Dênia Antunes Saúde-Guimarães, José Mário Carneiro Vilela, Margareth Spangler Andrade, Marta de Lana, Vanessa Carla Furtado Mosqueira

**Affiliations:** 1 Nucleus of Research in Biological Sciences (NUPEB), Biological Sciences Post-Graduation Program, Federal University of Ouro Preto, Minas Gerais, Brazil; 2 Laboratory of Pharmaceutics and Nanobiotechnology (LDGNano) – Pharmacy School, Federal University of Ouro Preto, Ouro Preto, Minas Gerais Brazil; 3 Post-graduation Program in Pharmaceutical Sciences (CIPHARMA), School of Pharmacy, Federal University of Ouro Preto, Ouro Preto, Minas Gerais Brazil; 4 Technological Center - CETEC SENAI - Minas Gerais Regional Department - Belo Horizonte, Minas Gerais, Brazil

## Abstract

Lychnopholide, a lipophilic sesquiterpene lactone, is efficacious in mice at the acute and chronic phases of Chagas disease. Conventional poly-ε-caprolactone (PCL) and long-circulating poly(D,L-lactide)-*block*-polyethylene glycol (PLA-PEG) nanocapsules containing lychnopholide were developed and characterized. Lychnopholide presented high association efficiency (>90%) with the nanocapsules. A new, fast and simple HPLC-UV-based bioanalytical method was developed, validated in mouse plasma and applied to lychnopholide quantification in *in vitro* release kinetics and pharmacokinetics. The nanocapsules had mean hydrodynamic diameters in the range of 100–250 nm, negative zeta potentials (−30 mV to −57 mV), with good physical stability under storage. Atomic force microscopy morphological analysis revealed spherical monodispersed particles and the absence of lychnopholide crystallization or aggregation. Association of lychnopholide to PLA-PEG nanocapsules resulted in a 16-fold increase in body exposure, a 26-fold increase in plasma half-life and a dramatic reduction of the lychnopholide plasma clearance (17-fold) in comparison with free lychnopholide. The improved pharmacokinetic profile of lychnopholide in long-circulating nanocapsules is in agreement with the previously reported improved efficacy observed in *Trypanosoma cruzi*-infected mice. The present lychnopholide intravenous dosage form showed great potential for further pre-clinical and clinical studies in Chagas disease and cancer therapies.

## Introduction

To date, benznidazole and nifurtimox are the only two available drugs to treat Chagas disease patients, both with high incidence of serious adverse effects and limited efficacy, especially at the chronic phase of infection^1^. Even though the disease has been known for more than a century, the search for new drugs and new formulations for the chemotherapy of Chagas disease remains an urgent challenge.

Lychnopholide (LYC) is a lipophilic sesquiterpene lactone isolated from *Lychnophora trichocarpha* Spreng. LYC presents a wide range of pharmacological effects^2, 3^, including activity against tumour cells *(in vitro*)^4^ and against trypanosomatides^5^.

Anti-trypanosomal efficacy *in vivo* was reported by our group^6, 7^. The efficacy of LYC loaded in polymeric nanocapsules (NC) was demonstrated for the first time in different strains of experimentally *Trypanosoma cruzi* (*T. cruzi)*-infected mice in the acute^6^ and chronic^7^ phases of the infection. In its free form and in NC formulation described herein, LYC was able to cure mice experimentally infected with *T. cruzi* strains sensitive, partially resistant and resistant to benznidazole at the acute^6^ and the chronic phases of the disease^6, 7^. Such efficacy had never been achieved by any other drug/formulation to date. In particular, to the best of our knowledge, no other molecule reported in the literature has been as effective at the chronic phase of infection with *T. cruzi* partially resistant strains. In the two above-mentioned studies, LYC in NC formulation was able to cure infected mice, whereas free-LYC only reduced the parasitaemia with no animal cure^6, 7^. As Chagas disease remains without an effective treatment, a detailed study of LYC as a potential new candidate for human Chagas disease therapy deserves investigation.

LYC has poor aqueous solubility and high lipophilicity^8^. No detailed studies of LYC formulation development suitable for oral and intravenous administration to humans have been reported to date. In addition, the absence of studies on LYC pre-formulation and comparative pharmacokinetics in mice, a suitable animal model to study *T. cruzi* infection and Chagas disease chemotherapy^9^, have been limiting its further evaluation. The ability of polymeric NC to improve the biopharmaceutical properties of lipophilic substances has been extensively demonstrated^10^. Recently, the association of LYC with polymeric NC was reported to modify the release properties of LYC *in vitro* ^8^, improve its efficacy in the acute^6^ and chronic phases of the experimental infection and prevent cardiotoxicity^11^ in mice by the intravenous and oral routes^7^.

Conventional polymeric NC are generally cleared rapidly from the blood circulation following opsonisation and phagocytosis and are passively targeted to the liver and spleen macrophages. This strategy reduces the ability of the active molecule to be delivered to other sites in the body or to remain in the bloodstream^12^. In contrast, long-circulating NC, which are surface-modified with covalently linked hydrophilic chains of polyethylene glycol (PEG) show slower recognition and uptake by phagocytic cells^13^ and exhibit longer plasma half-lives after intravenous administration^14^. The latter type of NC have higher chances of delivering lipophilic substances such as lychnopholide to the blood trypomastigote parasites that prevail in the acute phase of infection^15^. Furthermore, PEGylated NC may accumulate in the muscle cells of the heart and oesophagus, where amastigote intracellular forms of the parasite trigger severe cardiomyopathy and damage in the chronic phase of Chagas disease^15^. Such an accumulation of NC at the site of the parasite infection, which is characterized by leaky endothelium induced by inflammation, may be expected due to the *enhanced permeation and retention* (EPR) effect^16^, whereby PEGylated NC may selectively extravasate from the blood into surrounding inflamed tissues^17^.

This study reports the development of polymeric NC containing LYC, their physicochemical characterization and comparative pharmacokinetics in mice. Conventional NC were prepared from poly-ɛ-caprolactone (PCL) and compared with PEGylated NC prepared from poly(*D,L*-lactide)-*block*-poly(ethylene glycol) (PLA-PEG) designed for delayed blood clearance. We developed and validated a simple bioanalytical method based on high performance liquid chromatography with ultraviolet detection (HPLC-UV) for the quantification of LYC in plasma samples, applicable to perform *in vitro* release kinetic studies and to compare the pharmacokinetics of LYC in mice after intravenous administration of the formulations. The data reported here provide the foundations to explain and correlate the recently reported enhanced efficacy^6, 7^ and reduced toxicity^11^ of this new candidate in NC dosage form for the treatment of Chagas disease.

## Results

### Characterization of nanocapsules, LYC loading and entrapment percentages

Two types of NC containing LYC were developed in this work, namely conventional PCL NC and PEGylated PLA-PEG NC. The main physicochemical characterization data for both formulations are shown in Table 1. LYC was encapsulated at high entrapment percentages in both types of NC, higher than 87%. The highest values were obtained at a LYC loading of 2 mg/mL with PLA-PEG NC, reaching 98% entrapment. LYC loading in the carrier system was as high as 9 wt%. As LYC entrapment percentages were high, no further purification steps were necessary in the preparation process because the amounts of non-encapsulated drug were negligible.

**Table 1:** Physicochemical characterization of lychnopholide nanocapsules.

NC Formulation	LYC (mg/mL)	Mean^*a*^ hydrodynamic diameter ± SD (nm)	Polydispersity index^*a*^	Geometric diameters ± SD (nm)^*b*^ (AFM)	ζ potential ± SD (mV)^c^	Entrapment (%) ± SD^*d*^	LYC loading^*d*^ (wt%) ± SD
PCL NC	0.0	175 ± 14	0.14	215 ± 22	−40 ± 4	—	—
	2.0	183 ± 3	0.10	220 ± 31	−43 ± 4	94 ± 1	4.2 ± 0.8
	3.0	214 ± 6*	0.20	299 ± 49*	−53 ± 2*	90 ± 1	6 ± 1
	4.0	245 ± 10*	0.22	313 ± 69*	−57 ± 2*	93 ± 2	8 ± 1
PLA-PEG NC	0.0	109 ± 7	0.08	120 ± 28	−30 ± 4	—	—
	2.0	105 ± 7	0.10	130 ± 34	−37 ± 7	98 ± 2	5 ± 1
	3.0	138 ± 11*	0.13	150 ± 54	−43 ± 4*	94 ± 1	7 ± 1
	4.0	159 ± 8*	0.19	333 ± 77*	−40 ± 2*	87 ± 2	9 ± 2

^*a*^Mean of three measurements of three nanocapsule batches. Samples with values lower than 0.3 were considered monodispersed in size. ^*b*^Geometric diameters were measured on the height atomic force microscopy (AFM) images considering the width of the spheres at half height reported as the average of 60 particles and standard deviation. ^*c*^Measurement after dilution at 1:1000 in 1 mM NaCl (*n* = 3 batches). ^*d*^Determined by ultrafiltration/centrifugation method and assayed by HPLC following calculation described in the methods section. *Indicates significant differences compared to blank (no LYC) nanocapsules (*p* ≤ 0.05).

**Figure 1:**
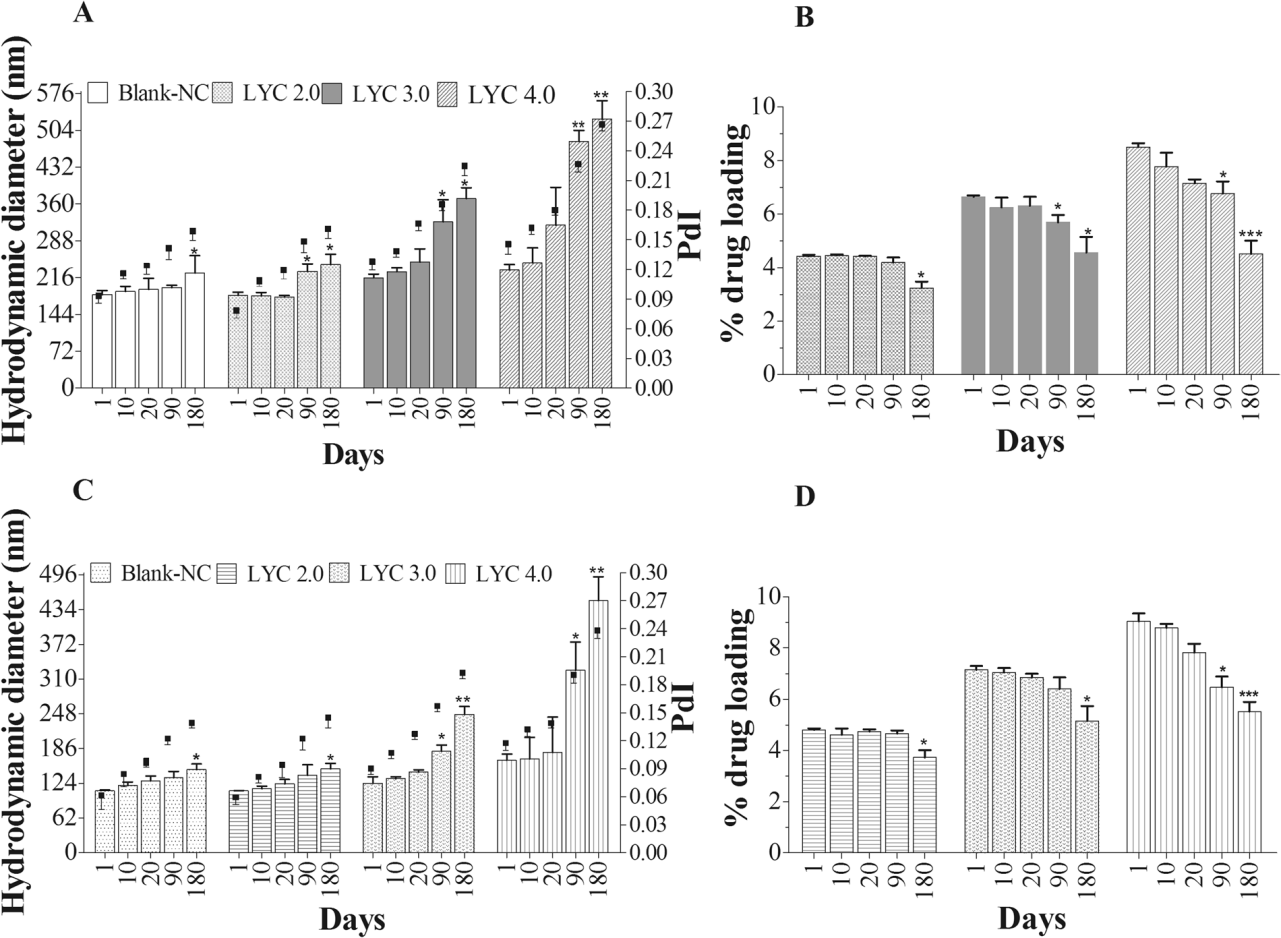
Physical stability of lychnopholide (LYC) nanocapsules of PCL (**A** and **B**) and PLA-PEG (**C** and **D**) up to 6 month storage at 4 °C. Mean hydrodynamic diameters are shown on the left axis and polydispersity index on the right axis (**A** and **C**); LYC loading (wt% ± SD) in graphs (**B** and **D**). Blank-NC are nanocapsules without LYC. ^*^Indicates 0.01 ≤ *p* < 0.05; ^**^indicates 0.001 ≤ *p* < 0.01; ^***^indicates *p* < 0.001 compared to day 1.

The mean diameters of the different NC formulations, as evaluated by dynamic light scattering (DLS), were not affected (*p* > 0.05) by LYC encapsulation at 2.0 mg/mL and were higher at LYC concentrations above 3.0 mg/mL, with a proportional increase in LYC entrapment percentages. All PLA-PEG NC were smaller in diameter (105 to 138 nm) than PCL NC (175 to 245 nm) (*p* ≤ 0.05). The polydispersity indices (PdI) lower than 0.3 with monomodal profiles for all NC indicate narrow size distribution and no differences between LYC-PCL NC and LYC-PLA-PEG NC or blank NC. The zeta potential analysis was used to detect changes in the surface charge of the particles due to the association of LYC to the NC. The NC formulations had negative surface charges, ranging from −30 to −57 mV. Values above 30 mV (in modulus) provide electrostatic repulsion between nanostructures and generally contribute to prolonged colloidal stability, as observed with our formulations in the experimental and storage periods (Table 1 and Fig. 1). The zeta potential of blank-PCL NC was significantly lower than that of blank-PLA-PEG NC. At LYC concentrations higher than 3 mg/mL the zeta potentials were lower (*p* > 0.05) compared to blank-NC for both formulations.

### Atomic force microscopy analysis

**Figure 2:**
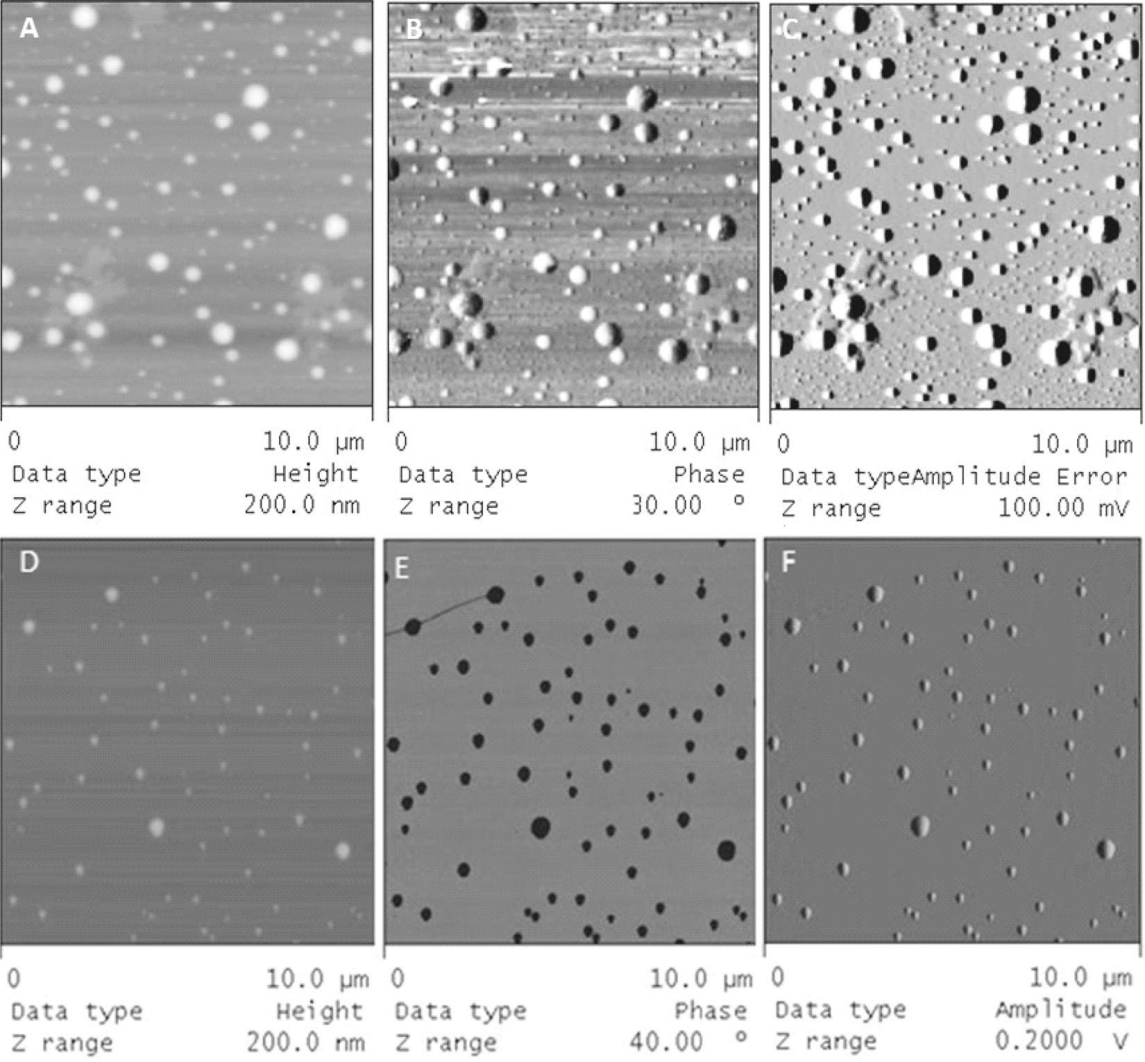
Atomic force microscopy images of lychnopholide nanocapsules obtained in *tapping mode* (10 × 10 µm scan size). LYC-PCL NC (top three images) and LYC-PLA-PEG NC (bottom three images) showing spherical nanostructures. Height (**A** and **D**), phase (**B** and **E**) and amplitude (**C** and **F**) images are shown.

The size and morphology of the NC were analysed by atomic force microscopy (AFM). The geometrical diameters of LYC-PCL NC and LYC-PLA-PEG NC studied by AFM increased with the percentages of LYC loading (Table 1 **)**, in agreement with the dynamic light scattering (DLS) data. However, the mean geometrical diameters were in general higher than the mean hydrodynamic diameters. The AFM images of all NC preparations evidenced nanostructures with a spherical shape (Fig. 2 **)**. PCL NC (Fig. 2A–C) showed more polydisperse and heterogeneous nanostructures in comparison with PEG-PLA NC (Fig. 2D–F). Small “micellar-like” spherical nanostructures (<40 nm) were observed on the AFM images of PCL NC, prepared with poloxamer, but were absent from PLA-PEG NC images. The mean geometrical size of PLA-PEG NC at 4.0 mg/mL of LYC (Table 1) increased relative to blank-NC (*p* ≤ 0.05), whereas no differences (*p* > 0.05) were evidenced between 2.0 and 3.0 mg/mL. All colloidal suspensions containing LYC associated to NC showed homogenous macroscopic aspect as a milky blueish opalescent colloidal suspension, without aggregation or signs of LYC crystallization.

### Development and validation of the bioanalytical method

We report a standardized, selective, precise and accurate HPLC-UV method for the quantification of LYC in NC formulations (PCL and PLA-PEG) and in plasma samples from mice treated with free LYC, LYC-PCL NC or LYC-PLA-PEG NC to apply in pharmacokinetic studies. This method is based on an analytical method used to evaluate LYC encapsulation efficiency previously reported by our group^8^ and adapted here to use single wavelength UV detection. Protein precipitation with acetonitrile was chosen as the best method for LYC extraction from plasma because it provides clean chromatograms and good percentages of recovery (99.8–103.8%). Under our experimental conditions, LYC eluted as a symmetrical peak, well separated from the solvent peak. Itraconazole (ITZ), used as the internal standard, presented good solubility in the mobile phase and a retention peak well separated from that of LYC, enabling calculation of LYC recovery. The method was selective considering that the chromatogram and the retention time of LYC (3.0 min) and of the internal standard (4.5 min) were not altered in the presence of the constituents used in PCL and PLA-PEG NC formulations, such as polymers, surfactants and oils (Supplementary Fig. SI 1). The limit of detection and limit of quantification values were 0.01 µg/mL and 0.5 µg/mL, respectively. The slopes, intercepts and the coefficients of determination were found to be y = (0.0509) x + (0.0036) and 0.9998 (*r* ^2^), respectively. The assay was linear between 0.5 µg/mL and 64 µg/mL in plasma, in spite of the small volume of blood collected (180–250 µL). The method showed suitable intra-run and intra-day accuracy and precision. The chromatographic conditions support short analysis times (6 min), adequate recoveries and no interference from the plasma matrix (supplementary information data). This method proves to be suitable for pre-clinical pharmacokinetic studies of the LYC NC administered in mice.

### Physicochemical stability of NC formulations under storage

The stability of LYC-loaded NC of PCL and PLA-PEG was monitored upon storage at 4°C for up to six months. The parameters determined at different time points were the particle diameter, polydispersity index (Fig. 1A and C) and LYC loading wt % (Fig. 1B and D). Both types of blank-NC had significant differences in sizes only after 180 days in comparison with the first day (Fig. 1). Significant differences in the parameters analysed were found from 90 days with either type of NC at a LYC concentration of 3.0 and 4.0 mg/mL. In general, an increase in LYC loading compromised NC stability for both NC types. All LYC-PLA-PEG NC were smaller and provided somewhat extended stability compared to LYC-PCL NC. The polydispersity indices remained below 0.3 and the formulations maintained their monodispersed character during the evaluation period. LYC association with both types of NC at 2.0 mg/mL produced stable formulations for up to six months under our experimental conditions (Fig. 1 **)**. Therefore, further *in vitro* and *in vivo* studies were carried out at concentrations up to 2 mg/mL. Both NC types, with PEG physically adsorbed to the particle surface, as in PCL NC, or covalently linked as in PLA-PEG NC, showed to be physically (colloidal stability) and chemically stable (no LYC degradation or reduction in loading) for up to six months at 4°C at approximately 4–5 wt% loading. No sediment, creaming, oil leakage, polymer aggregation or flocculation of the formulations was observed during this period. In addition, macroscopic LYC precipitates or microscopic crystals were not observed under storage.

### Lychnopholide release profiles *in vitro*

**Figure 3:**
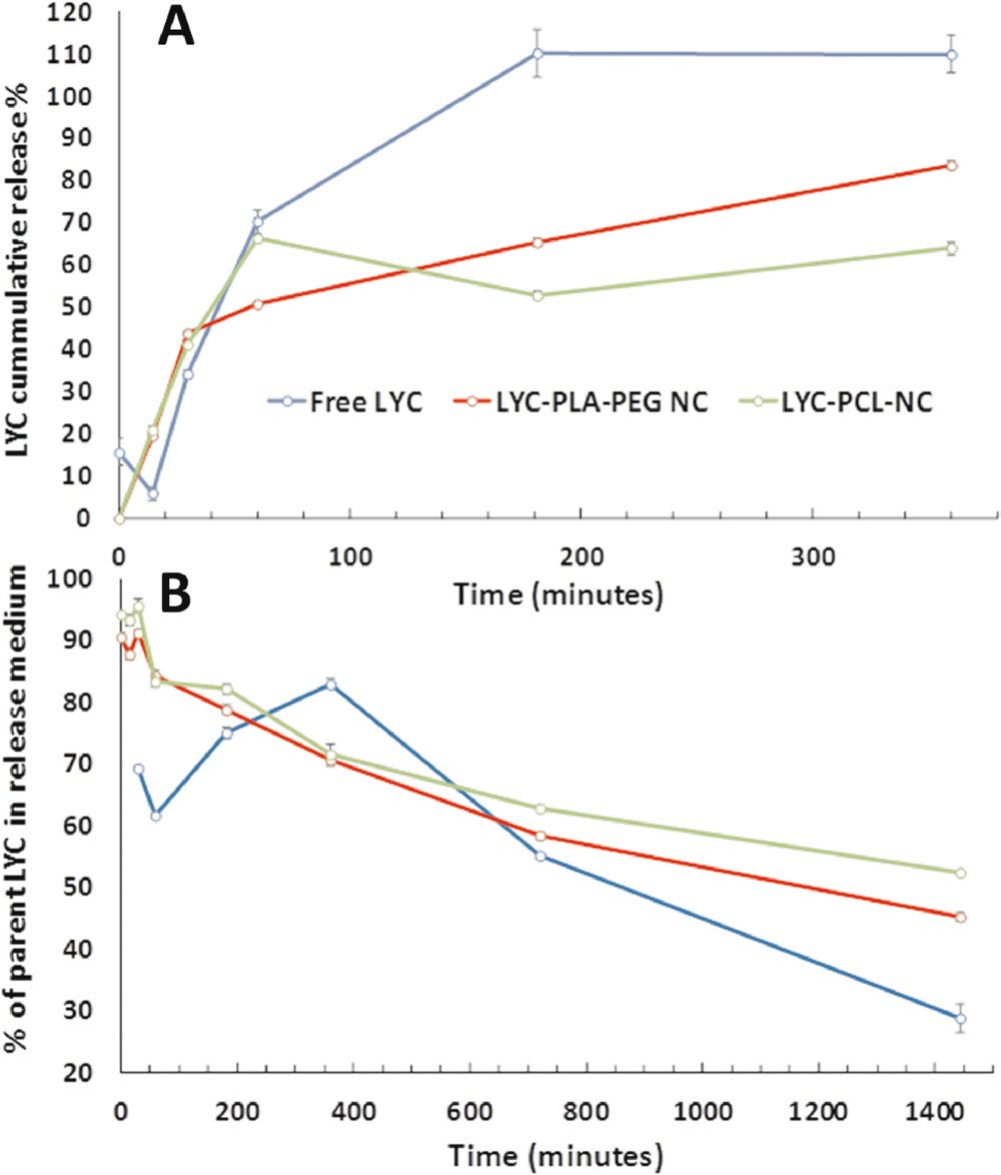
Lychnopholide (LYC) dissolution and release profiles from nanocapsules (graph **A)** and percentage of parent lychnopholide (graph **B**) determined in phosphate buffered saline (pH 7.4) with 40% v/v of mice plasma. Parent LYC is the LYC content determined in the release medium divided by the total LYC placed in the release medium × 100. Metabolites or degradation products were neither identified nor assayed.

The dissolution profile of LYC in PBS/mice plasma showed a slow rate of dissolution and even after two hours some crystals were still visually observed in the medium. The NC loaded with LYC at 1 mg/mL dispersed very fast in the medium, and both NC presented a biphasic release profile, with an initial “burst” release in the first 60 min, followed by a slower “sustained” release for PLA-PEG NC (Fig. 3A), as often observed for polymeric NC^18^. The LYC release rates were similar for both NC in the first 30 min. After 60 min the LYC release from PCL NC reached a “plateau” at approximately 64% whereas PLA-PEG NC maintained a constant release rate, offering better control up to 83% after 360 min. The percentage of LYC remaining in the release medium containing plasma at 37°C, expressed as the fraction of LYC detected in the medium at each time point divided by the initial LYC concentration, decreased rapidly with time, indicating chemical degradation (Fig. 3B). However, the rate of LYC degradation was dependent on the type of formulation. Hence, after 1440 min the parent LYC concentration had decreased by 72%, 48% and 55% for free-LYC, PCL-NC and PLA-PEG-NC, respectively (Fig. 3B). Free-LYC complete dissolution was achieved within 180 min, after which a higher rate of degradation in the medium was observed. Degradation was confirmed by the appearance of new peaks in the chromatogram (Supplementary Fig. SI-2). The identification of degradation products or metabolites was not the focus of this study.

### Lychnopholide pharmacokinetics in mice

**Figure 4:**
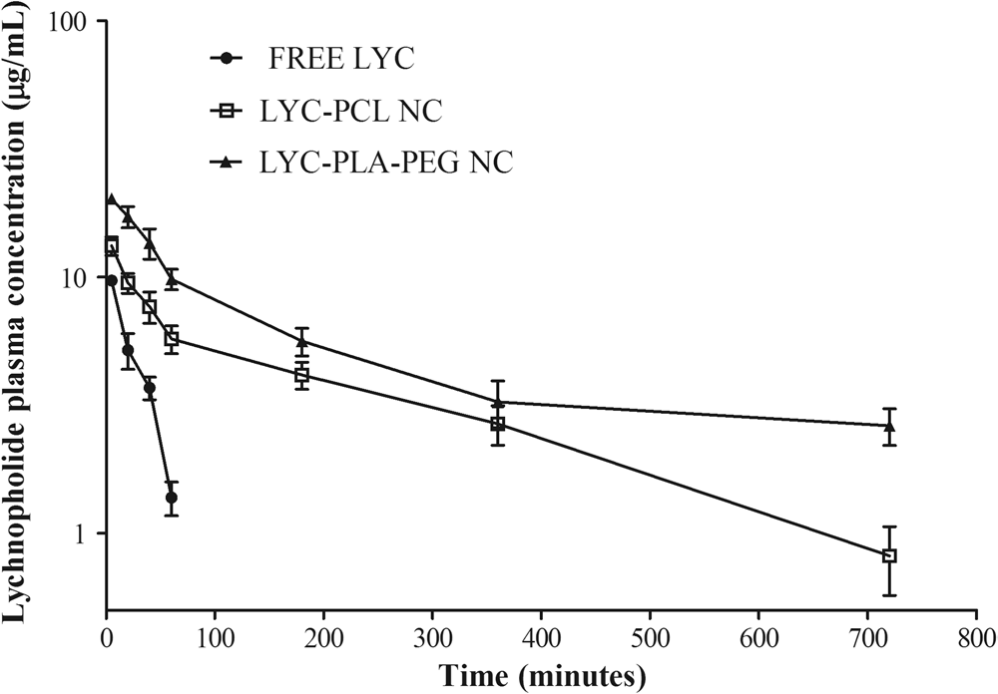
Lychnopholide (LYC) plasma concentration–time profiles after intravenous administration (*bolus*) in mice. Free LYC (*iv* solution), LYC-PCL NC and LYC-PLA-PEG NC were administered at a single dose of 12.6 mg/kg in mice (mean ± standard deviation, *n* = 6).

Lychnopholide was detected in the plasma of mice up to 60 min after injection for free-LYC solution and up to 720 min for both types of LYC NC after a single intravenous LYC dose of 12.6 mg/kg. Plasma samples were also collected at 1080 and 1440 min, processed and analysed, but LYC concentrations were below the limit of quantification at these time points. LYC plasma concentration–time profiles were obtained and compared after the administration of free LYC, LYC-PCL NC and LYC-PLA-PEG NC to mice. No acute signs of toxicity (piloerection, ataxia, dyspnoea or convulsion) were observed after the administration of this single dose. The areas under the curves and the plasma clearance profiles were substantially different (p < 0.05) for free LYC solution, PCL NC and PLA-PEG NC (Fig. 4). There was a drastic decrease in LYC plasmatic clearance after encapsulation (p < 0.05), indicating that the polymeric NC were able to retain LYC in the blood compartment for longer times **(**Table 2 **)**. There were also significant differences between the plasma concentrations of LYC encapsulated in LYC-PLA-PEG NC and LYC-PCL NC (Fig. 4). However, within the first 360 min after injection their general plasma clearance profiles were similar (Fig. 4). The analysis of the pharmacokinetic parameters of LYC-PCL *versus* free LYC showed a 7.5-fold higher AUC_0-∞_ for LYC-PCL NC (Table 2). PLA-PEG NC induced an increase in the AUC_0-∞_ of 16-fold compared to the free-LYC. The MRT of LYC associated to PCL and PLA-PEG NC showed a 11 and 24-fold increase compared to free LYC, respectively. In addition, the LYC half-lives for PCL and PLA-PEG NC were 10-fold and 26-fold higher than that for free-LYC (21 min), respectively. Furthermore, the corresponding total body clearance decreased dramatically (17-fold) with LYC associated to PLA-PEG NC. There was an increase in the AUC_0-720_ for LYC-PLA-PEG NC of 1.6-fold compared to PCL NC. The *t*_1/2_ value for LYC-PLA-PEG was 2.4-times higher than that for LYC-PCL NC.

**Table 2:** Lychnopholide (LYC) pharmacokinetic parameters following intravenous (*bolus*) administration of different nanocapsule (NC) formulations.

Pharmacokinetic parameters	Formulations		
	Free-LYC	LYC-PCL NC	LYC-PLA-PEG NC
AUC_0-720_ (µg·min/mL)	304 ± 32^*a*^	2380 ± 358^*^	3715 ± 561^*#^
AUC_0-∞_ (µg·min/mL)	355 ± 35	2648 ± 443^*^	5756 ± 1017^*#^
MRT_0-∞_ (min)	28 ± 2	299 ± 24^*^	681 ± 32^*#^
Clearance (mL/min)	39 ± 4	4.7 ± 0.8^*^	2.3 ± 0.3^*#^
V_*d*_ (mL)	1163 ± 195	1547 ± 113^*^	1755 ± 233^*^
*t*_½_ (min)	21 ± 1	227 ± 22^*^	538 ± 18^*#^
*λ*_*z*_ (min^−1^)	0.033 ± 0.002	0.0031 ± 0.0004^*^	0.00129 ± 0.00004^*#^

^*a*^AUC_0-60_ (µg·min/mL) for free-LYC. Dose 12,600 µg/kg mouse bodyweight. MRT: mean residence time; V_*d*_: volume of distribution; *t*_½_: plasma half-life. *λ*_*z*_: elimination constant. Terminal elimination slope was calculated based on the last three data points. ^*^p < 0.05 compared to free LYC; ^#^p < 0.05 compared to LYC-PCL NC.

## Discussion

Nanoprecipitation is a fast and simple method for the preparation of NC and offers advantages for intravascular delivery due to the use of biodegradable polymers and non-chlorinated solvents, low toxicity excipients and low nanoparticle sizes^19^. The lipophilic LYC was efficiently loaded in NC at different concentrations with high loading percentages up to ~9 wt%, probably due to the affinity of LYC for the NC triglyceride liquid core. In our case, no purification step was necessary to remove non-encapsulated LYC due to high entrapment efficiencies (>90%). The drug loading gives an insight into the capacity of the NC to transport the payload, indicating a suitable nanocarrier design to transport LYC by the intravenously route. In addition, monodispersed NC populations were obtained, with sizes between 100 and 250 nm without aggregates and with zeta potential values, which may have contributed to their colloidal stability under storage. LYC had little influence on the NC surface charge up to 4–5 wt% w/w loading. Above this concentration, the zeta potential values (in modulus) and mean sizes increased significantly. This may be attributed to a high LYC association to the inner oily core of NC. As LYC loading increases, its saturation and subsequent association with the polymeric wall or with the surface of the NC increases, affecting the equilibrium of polarizable groups at the NC surface. However, even in this situation, little influence on the NC colloidal stability was observed in the short storage time (90 days). At suitable LYC loading up to 2 mg/mL, the NC maintained stability of the active content in aqueous suspension. Nonetheless an additional step to freeze-dry the NC would deserve investigation to further extend the product shelf-life.

The reduced size of the PLA-PEG NC compared to PCL NC may be attributed to differences in the interfacial tension induced by the use of different polymers, the more hydrophobic PCL or the amphiphilic diblock PLA-PEG polymers, which during the mixing of the solvents generate NC of smaller size in the latter case^20^. In AFM analysis, we observed NC flattening upon tip pressure during scanning, as previously reported^21^. Therefore the diameters of NC by AFM are in general overestimated in comparison with hydrodynamic diameters by DLS. The AFM technique showed clearly that the presence of the poloxamer surfactant induced some degree of polydispersity and heterogeneity in the PCL NC formulation that were not visible on images of PLA-PEG NC (Fig. 2), as already discussed elsewhere^21^. The heterogeneity in PCL NC may be expected to result in complex *in vitro* release and *in vivo* behaviour due to the participation of structures of different nature and sizes^22^. This heterogeneity may therefore have contributed to an uneven release profile of LYC from PCL NC (Fig. 3A). Oppositely, PLA-PEG NC showed low dispersity in size by both DLS and AFM techniques and better control over the release rate of LYC *in vitro*.

An interesting aspect reported in our study is the LYC degradation in mouse plasma, particularly after 180 min incubation, and the relative protection against degradation provided by the NC (Fig. 3B), as similarly observed with camptothecin loaded in PLA-PEG NC^23^. This is also in line with our previous report that PCL NC were able to protect omega-3 fatty acid from oxidation and maintain *in vitro* anti-proliferative effects against breast cancer cells^24^. Previous *in vitro* release studies demonstrated that LYC is slowly released from PCL NC in phosphate-buffered saline (PBS) with 0.5% Tween for 2880 min^8^. However, 40% of mouse plasma in the medium induced a much faster release (80% in 360 min, this study, *vs* approx. 12% in PBS/Tween^8^), probably because proteins act as LYC acceptors favouring LYC partition to the external aqueous medium from the triglyceride-based core of the NC. The main driving force is probably the LYC strong binding to mouse plasma proteins as recently reported with rat plasma^25^. This accelerating effect of plasma proteins on the release of a lipophilic drug has already been observed with similar PLA-PEG NC^26, 27^. However, we observed an unexpected degradation of LYC over time in mouse plasma medium. Cholinesterases and arylesterases are esterases involved in drug hydrolysis in plasma^28^ and may be responsible for LYC degradation due to the presence of labile lactone and ester groups in its chemical structure. The role of esterases in the release of dexamethasone-palmitate ester from lipid nanoparticles was confirmed in 10% mouse plasma by using an esterase inhibitor that reduced significantly the rate of drug release^29^. Esterases could potentially accelerate polyester-based NC wall degradation during our experimental conditions. However, there is evidence for the stability of our NC during the release experiment. Indeed, both NC formulations decreased significantly the rate of LYC degradation for up to 1440 min. In addition, DLS measurements confirmed the presence of the NC at 1440 min in the release medium, with diameters of 151 and 164 nm and PdI of 0.25 and 0.13 for PLA-PEG and PCL NC, respectively, close to the values determined for the parent formulations. Thus, *in vitro* release in medium containing plasma is more realistic with nanoformulations intended to be administered by the intravenous route^30^. Another important observation is better control over the release kinetics of LYC with PLA-PEG NC than with PCL NC in the plasma of mice *in vitro*. Similar results were obtained with other lipophilic molecules encapsulated in PLA-PEG NC^26, 27^. It is expected that covalently linked PEG provides more stable shielding of the NC surface reducing interaction of the carrier with proteins^31^. However, abundant studies in the literature have shown that protein corona is formed *in vivo* even with nanostructures presenting PEG dense brushes at the NC surface^32^. Under our experimental conditions PEG chains in PLA-PEG NC were not able to shield the negatively charged groups of the lecithin at the NC surface, as indicated by the negative zeta potential values, probably because the PEG chains are present at low surface densities.

Our bioanalytical method to quantify LYC in mice plasma is simple, selective, accurate and reproducible. Recently, a very sensitive method was reported using liquid chromatography–tandem mass spectrometry (LC-MS/MS) to quantify LYC in rat plasma^33^ that was applied to LYC pharmacokinetic analysis^25^. Although our method was less sensitive because we used UV detection, we found a better LYC recovery from mouse plasma (~98%) using the protein precipitation method compared to liquid-liquid extraction (63%) previously reported^33^. Furthermore, UV detection is cost-effective and more accessible than mass spectrometry in developing countries, where LYC monitoring in plasma may be useful in pharmacokinetic/pharmacodynamics studies with *T. cruzi*-infected models, in pre-clinical and clinical phases. The mouse is the animal species classically used to study experimental *T. cruzi* infection, including chemotherapy of Chagas disease and evaluation of new compounds against this disease, with similar characteristics to human infection^9^. Thus, we used mice in our pre-clinical evaluation of LYC pharmacokinetics to further correlate with reported efficacy data using the same animal model^6, 7^. Non-compartmental pharmacokinetic analysis was used to compare LYC exposure after *iv* administration of the different formulations. The pharmacokinetic study was conducted at a single dose of 12.6 mg/kg administered intravenously in healthy mice. This dose was chosen based on a preliminary pilot study whereby three doses (2.0, 6.0 and 12.6 mg/kg) were administered *iv* to mice and LYC determined in the plasma at 60 min and the criterion was LYC quantification in the blood. In the present study we found that free LYC distributes very fast to tissues and is metabolized or degraded after intravenous administration with high plasma clearance rates. Recently, intravenous and oral pharmacokinetics of LYC were determined in rats^25^ and fast clearance of LYC was also observed. LYC high lipophilicity (clog P > 5) indicates high biological membrane permeability and some drawbacks, such as a short drug half-life, which was confirmed in our present study and also in intravenous pharmacokinetics in rats^33^. The NC improved dramatically the body exposure to LYC (higher AUC) and LYC clearance was drastically reduced (16-fold) with PLA-PEG NC. This data corroborates our previous studies of higher efficacy of LYC-PLA-PEG NC compared to free-LYC (100% *vs* 0% cure, respectively) in the acute phase of experimental Chagas disease in Y strain *T. cruzi*-infected mice^6^. The long-circulating property of PEGylated NC compared to free LYC is useful in the acute phase of Chagas disease, where *T. cruzi* trypomastigotes circulate in the blood compartment.

Despite a slight improvement of the pharmacokinetic parameters of LYC-PLA-PEG NC compared to LYC-PCL NC, the pharmacokinetic profiles of both NC were similar and both increased LYC blood exposure, which indicates that both NC provide control over the release of LYC and may prevent or limit LYC degradation. It has been shown that conventional NC stabilized by PEG-containing surfactants, including poloxamer, used in PCL NC formulation, efficiently increase the circulation times of encapsulated lipophilic compounds^34^.

In order to reach the leaky endothelium of inflamed tissues infected by *T. cruzi*, long circulating NC with sizes lower than 200 nm, such as the ones in this study, are of particular interest. As shown by de Mello *et al*. NC containing LYC also showed efficacy in the chronic phase, curing 50% and 33% of mice chronically infected with the *T. cruzi* Y strain, for PLA-PEG and PCL NC, respectively^7^. This difference in efficacy is supported by the pharmacokinetic behaviour of LYC in NC, including a slightly better performance of PLA-PEG NC than PCL NC. PLA-PEG NC provided similar improvement in efficacy of halofantrine in comparison with conventional PLA NC against *Plasmodium berghei*-infected mice and efficacy also correlated with increased AUC^35^. LYC pharmacokinetic analysis shows clearly that sustained LYC plasma levels may be achieved by nanoencapsulation. However, in the plasma LYC may remain associated to NC for longer periods and be only partially released. This assumption is in agreement with the low cardiotoxicity of LYC in NC formulation^11^. In addition, the total LYC plasma levels do not necessarily represent the levels of free LYC available to exert pharmacological activity, considering that part of the LYC detected in the plasma can be associated to plasma proteins or remain encapsulated, as suggested by our release experiment. Our pharmacokinetic results are consistent with a probable improvement of LYC biodistribution profile when administered in NC dosage form, which would allow the parasites in the tissues to be reached. As the body exposure to LYC increases when administered in NC dosage form, a detailed investigation of LYC potential side effects on the cardiovascular system was presented, complementing the present work^11^. Furthermore, as LYC is also active against tumour cells^4^, a general toxicity profile should be evaluated in future pre-clinical studies.

## Conclusion

Biodegradable polymeric NC containing LYC were prepared and characterized. Encapsulation of LYC was achieved at high payload and the NC were stable upon storage and with sizes suitable for intravenous administration. PLA-PEG NC effectively controlled the release of LYC in the plasma of mice and increased drastically body exposure. Even though the best performance was obtained with PLA-PEG NC, PCL NC also showed promising results. In addition to improving LYC pharmacokinetic parameters (*iv*), the NC protected the encapsulated LYC from degradation in mouse plasma. The pharmacokinetic analysis of LYC after intravenous administration in healthy mice correlates well with LYC efficacy *in vivo*. Particularly, PEGylated NC may be beneficial to the treatment of trypanosomatid-based infectious diseases and potentially in cancer chemotherapy.

## Methods

### Materials

LYC isolation from *L. trichocarpha*, purification and characterization steps were carried out in our group as previously reported^8^. PLA-PEG [poly(*D,L*-lactide)-*block*-polyethylene glycol average M_w_ 49,000 g/mol with a PEG block of M_n_ 5,000 g/mol] was obtained from Alkermes (Cambridge, USA) and used without further purification. Polymer poly-*rac-*lactide (PLA) (Resomer R203 S, inherent viscosity 0.25–0.35 dL/g, Mw 18,000–28,000 g/mol, ester terminated) was provided by Boehringer Ingelheim (Germany). PCL with average M_w_ 42,500 g/mol, Poloxamer^®^188, Tween^®^80, ethylenediaminetetraacetic acid (EDTA) and itraconazole standard (ITZ) ( ± )-1-sec-butyl-4-[p-[4-[p-[[(2 R,4 S)-2-(2,4-dichlorophenyl)-2(1H-1,2,4-triazol-1-lmethyl)-1,3-dioxolan-4-yl]methoxy]phenyl]-1-piperazinyl]phenyl D2-1,2,4-triazolin-5-one were purchased from Sigma-Aldrich (Brazil). Medium chain triglyceride, Miglyol^®^810 N (capryc/caprylic triglyceride) was provided by Hulls (Germany). Soy lecithin with ~70% phosphatidylcholine (Epikuron^®^170) was provided by Cargill (Le Blanc Mesnil, France). HPLC grade acetonitrile, methanol, ethyl acetate, *N,N*-dimethylacetamide and analytical grade dimethyl sulfoxide were provided by Tedia (Rio de Janeiro, Brazil). Analytical grade polyethylene glycol 300 (PEG 300) and acetone were obtained from Vetec (Rio de Janeiro, Brazil). A Symplicity^®^ System (Millipore, Bedford, USA) was used to produce ultrapure water (18.2 MΩ·cm).

### Preparation of lychnopholide nanocapsules and intravenous solution

PCL NC were prepared by the interfacial polymer deposition followed by solvent displacement method^19^. Briefly, PCL NC were prepared by dissolving 80 mg of PCL in a solution of acetone (10 mL) containing 40 mg of Epikuron, 250 µL of Miglyol and LYC (20 to 40 mg). This organic solution was poured into a solution of ultrapure water (20 mL) containing 75 mg of Poloxamer, under magnetic stirring. The final colloidal suspension was maintained under magnetic stirring for 10 min, the solvents were evaporated under reduced pressure (Laborota 4000, Heidolph Instruments, Germany) to a volume of 10 mL containing NC with final LYC concentrations of 2 to 4 mg/mL. Long-circulating, PLA-PEG NC containing LYC were prepared replacing PCL by a 1:1 mixture of PLA-PEG (60 mg) and PLA (60 mg) and in the absence of Poloxamer in the aqueous phase. Blank-NC were obtained in the absence of LYC. No purification steps were needed in both cases, because no polymer aggregation, LYC crystallization or non-encapsulated LYC were found in significant concentration in the aqueous external phase of the NC colloidal suspension (see results section). LYC solution (free LYC) for intravenous administration was prepared by dissolving 4 mg of LYC in 100 µL of a mixture of *N,N*-dimethylacetaminde and PEG 300 at 40:60 (v/v) and further diluting in 5% (w/v) glucose to reach the correct dose (12.6 mg/kg) at a final volume of 200 µL for intravenous injection in mice.

### Characterization of lychnopholide-loaded nanocapsules

#### Size distribution and zeta potential

The mean hydrodynamic diameter of NC and particle size polydispersity (PdI) were determined by DLS on a Beckmann Coulter (Fullerton, USA) Nanosizer N5Plus analyser at a scattering angle of 90° at 25°C. Samples were analysed after 1:1000 dilutions in 0.22 µm filtered ultrapure water. The zeta potential (ζ) was determined by DLS coupled to microelectrophoresis on a Zetasizer HS3000 (Malvern Instruments, Malvern, UK) with sample dilution 1:1000 in 1 mM NaCl 0.22 µm filtered. Ten measurements were recorded in triplicate for each sample and processed using multimodal analysis. Values reported in Table 1 are the means ± standard deviation (SD) of at least three different batches of each NC formulation.

### Atomic force microscopy analysis

NC samples were analysed by scanning probe microscopy in atomic force microscopy (AFM) mode. Analyses were performed in air at room temperature, on a Dimension 3000 equipment monitored by a Nanoscope IIIa controller from Digital Instruments (Santa Barbara, CA, USA). A droplet (5 µL) of NC suspension was deposited on freshly cleaved mica, spread and dried under a stream of argon gas. The images were obtained in *tapping* mode, using commercial Silicon probes, with cantilever having a length of 228 µm, resonance frequencies 75–98 kHz, spring constants of 3.0–7.1 N/m and a nominal tip curvature radius of 5 nm. The scan rate was 1 Hz. Dimensional analyses were performed with the Nanoscope 5.31r1 “section analyses” software. Geometrical diameters were measured on the height images considering the width of the spheres at half height and are reported as the average of values obtained from 60 particles.

### Chromatographic conditions for lychnopholide assay

The HPLC system used to quantify LYC consisted of a Waters Alliance E2695 separation module, autosampler, pump, column oven and Waters 2489 UV detector set at 267 nm (Waters Corporation, Milford, MA). The separation was performed using a 150 mm × 4.6 mm C18 Gemini-NX Phenomenex^®^ column with a 5 µm particle size and protected by a Gemini-NX C18 Phenomenex^®^ security guard pre-column (2 mm × 4.6 mm, 3 µm). The mobile phase, acetonitrile: water (70:30 v/v), was prepared daily, filtered through a 0.45 µm membrane and degassed in an ultrasonic bath before use. Chromatographic separation was carried out at a 1.0 mL/min flow rate with isocratic elution at 30°C column temperature. The injection volume was 25 µL for all samples, standards and controls.

### Determination of LYC loading and entrapment percentages

The LYC content in NC of PCL and PLA-PEG formulations were analysed using the method previously published^8^ and slightly adapted to improve sensitivity with the UV detector set at 267 nm instead of using a photodiode array detector. The fraction of LYC in the external phase of the colloidal suspension was assessed by ultrafiltration in an Amicon Ultra centrifugal filter (50,000 Da Millipore^®^) of 400 µL of total NC suspension. The device was centrifuged at 500 × *g* for 30 min. The ultrafiltrate (100 µL) was vortex-mixed with 400 µL of acetonitrile, centrifuged and the concentration of LYC in the supernatant quantified by HPLC. LYC associated with the NC was retained in the upper compartment of the device. To determine the total LYC content in the final NC suspension, 100 µL of the NC suspension was added to 900 µL of acetonitrile to disrupt the NC (all polymers and LYC are soluble in acetonitrile), vortex-mixed for 5 min (Vortex Instrument, IKA, Germany) and centrifuged (Centrifuge 5415 D, Eppendorf, USA) and the supernatant analysed. All samples were filtered (0.45 µm) before injection and 25 µL were injected for HPLC analysis. The analyses were performed in triplicate. LYC loading (w/w %) was calculated as the ratio of the mass of LYC encapsulated in NC by the total mass of all raw materials in the NC preparation^36^. The LYC entrapment % was calculated as the ratio of the mass of drug associated with the NC (calculated from the difference between the total LYC content quantified in the final colloidal suspension and the free LYC in the external aqueous phase quantified in the ultrafiltrate by HPLC) by the mass of LYC used in formulation × 100.

### Physicochemical stability of nanocapsule formulations under storage

The physicochemical stability of the NC in terms of mean diameter, polydispersity and LYC content (w/w %) in the different NC formulations was assessed by storing aqueous NC dispersions in sealed glass bottles at 4 °C protected from light, during a 6-month period. Stability was evaluated by comparing the variations from the mean NC hydrodynamic diameters, PdI and LYC loading as a function of time.

### Development and validation of the bioanalytical method

Stock solutions of LYC and ITZ internal standard in acetonitrile were prepared at concentrations of 1000 µg/mL and 250 µg/mL, respectively, and stored at −20 °C. The working solutions were prepared at concentrations of 0.25, 0.50, 0.75, 1.0, 2.0, 4.0, 8.0, 10.0, 16.0, 32.0 and 64.0 µg/mL. The specificity of the bioanalytical method was determined by comparing the chromatograms of blank mouse plasma (six different animals) spiked with LYC (10 µg/mL) and ITZ (2.5 µg/mL) to ensure no interference from biological matrices at the retention time of ITZ (4.499 min) and LYC (2.946 min), respectively (Supplementary Material SI-1). Linearity was determined by least-squares linear regression over the concentration range of 0.50–64 µg/mL of the curve plotted from the ratio of LYC by the internal standard peak areas versus concentration of calibration standards using triplicates.

### Lychnopholide extraction from biological samples

Blood samples (250 µL) were collected from mice in polypropylene tubes containing EDTA 0.18% w/v. The plasma was separated by centrifugation at 400 × *g* for 3 min at 4 °C, aliquoted and stored at −80 °C until analysis. To precipitate plasma proteins during LYC extraction acetonitrile (900 µL) was added to 90 µL plasma aliquots spiked with 10 µL of IS solution (250 µg/mL). The samples were vortex-mixed for 2 min and centrifuged at 9,300 × *g* for 10 min. The supernatant was collected, filtered on 0.45 µm PTFE 4 mm syringe filters (Millipore, USA) and evaporated to dryness in a nitrogen-stream apparatus (TE-019 Concentrator/Tecnal, Brazil). Each sample was reconstituted in 100 µL of the mobile phase or further diluted when necessary to enter the linear range of the calibration curve before injection in the HPLC system in triplicates (25 μL). All the validation parameters and the results are described in detail in the **supplementary material** available.

### *In vitro* lychnopholide release kinetics in mice plasma

The release of LYC from the different formulations in mice plasma was determined following the previously described protocol^26^. Briefly, LYC in its free form (1 mg of LYC crystals) or 1000 µL of PCL or PLA-PEG NC suspension at LYC concentration of 1 mg/mL were placed in tubes containing 5 mL of release medium composed of phosphate buffered saline pH 7.2 with 40 v/v % of mice plasma, previously equilibrated at 37°C in a thermostated shaker bath. At time 0, the media were vortexed and a 100 µL sample was collected to determine the total amount of LYC. At each time point (15, 30, 60, 180, 360, 720, 1440 min) two samples were withdrawn, one for total LYC determination in total media (100 µL), and another (400 µL) for ultrafiltration (600 × *g* for 5 min) in an Ultrafree^®^MC 0.1 µm pore centrifugal device (Millipore^®^) to determine LYC released in the aqueous medium including that bound to plasma proteins. Acetonitrile (900 µL) was added to 100 µL of the ultrafiltrate and total medium samples, the samples were vortex-mixed, centrifuged at 400 × *g* for 5 min elaborated as described in the bioanalytical methodology and assayed by HPLC. This experiment was carried out in triplicate.

### Preclinical LYC pharmacokinetics in mice

#### Animals

Female Swiss albino mice were used and maintained according to guidelines established by Sociedade Brasileira de Ciência em Animais de Laboratório (SBCAL). The mice were aged 28–30 days, weighing 20–25 g, to allow correlation with previous studies on therapeutic efficacy of free-LYC and LYC in NC as described by Branquinho *et al*.^6^ and Mello *et al*.^7^. The experiments were approved by the Ethical Committee on Animal Experimentation of the Universidade Federal de Ouro Preto, Brazil (protocol n° 2010/13). The mice were maintained in a specific pathogen free room at 20–24 °C under a 12/12 h light/dark cycle and were provided with sterilized water and chow *ad libitum*.

Free LYC as an intravenous solution, PCL NC and PLA-PEG NC at a LYC dose of 12.6 mg/kg of bodyweight were administered in bolus by intravenous injection in the mice tail vein (125–160 μL according to animal bodyweight of 2 mg/mL of LYC-NC) as a single dose to three groups of healthy mice. Even though all formulations were monodisperse and had mean diameters below 250 nm, they were filtered through a 0.45 μm sterile filter (Millipore) before intravenous injection. At each time point of 5, 20, 40, 60, 180, 360, 720, 1080, 1440 min after administration blood was collected from six animals (n = 6) through the orbital sinus vein (150–250 μL) in tubes containing EDTA (0.18% w/v) and the animals were sacrificed. The blood samples were centrifuged (400 × *g*) for 4 min at 4°C to separate the plasma. The plasma samples were stored at −80°C until analysis. LYC was extracted from the plasma samples by the protein precipitation method described above.

### Pharmacokinetic parameter calculations

The pharmacokinetic parameters for LYC were determined by means of non-compartmental approach based on the plasma-concentration profiles of free-LYC in solution, in PCL NC and in PLA-PEG NC formulations after *bolus* intravenous administration. The area under the curve (AUC_0-t_) and the area under the first-moment curve (AUMC_0-t_), where *t* is the last sampling time were calculated by the linear trapezoidal rule method from time zero to the 12 h sample^37^. The area under the curve extrapolated to infinity (AUC_0-∞_) was obtained by adding C_p_/*λ*_z_ to AUC_0-12h_, where C_p_ is the last measurable concentration. *λ*_z_ is the terminal elimination rate constant determined by linear least-square regression through the last three plasma-concentration time points for all formulations. The elimination half-life (*t*_1/2_) was calculated as 0.693/*λ*_z_. The AUMC_0-∞_ was calculated as AUMC_0-t_ + (C_p_ × t/ *λ*_z_) + (C_p_/ *λ*_z_^2^). The clearance (*Cl*) was calculated as Dose/AUC_0-∞_. The apparent volume of distribution (V_*d*_) as MRT × *Cl*, where MRT is the mean residence time obtained by AUMC_0-∞_/AUC_0-∞_. Data analysis was performed with PKSolver^®^ add-in software (Microsoft^®^ Excel)^38^. Mean ± SD at each time point (n = 6) were plotted in plasma × concentrations curves.

### Statistical analysis

The significant differences in hydrodynamic diameters, zeta potential values between each experimental group were performed by ANOVA and by unpaired two-tailed *t*-test. Newman-Keuls Multiple Comparison test was performed to compare pharmacokinetic data with GraphPad Prism version 5.01 (GraphPad Software, San Diego, CA). The level of significance was set at *p* < 0.05 with 95% confidence interval.

### Data availability

The datasets generated during and/or analysed during the current study are available from the corresponding author on reasonable request.

## Electronic supplementary material

Supplementary information
