# Supplementary material for: Increased Body Exposure to New Anti-Trypanosomal Through Nanoencapsulation
Source: Sci Rep. 2017 Aug 16;7:8429. doi: 10.1038/s41598-017-08469-x (PMC5559493; doi:10.1038/s41598-017-08469-x)
Supplement: Supplementary file 1 — Supplementary information [file 41598_2017_8469_MOESM1_ESM.pdf]

Supplementary Information

INCREASED BODY EXPOSURE TO NEW ANTI-TRYPANOSOMAL THROUGH NANOENCAPSULATION

Renata Tupinambá Branquinho[a,b], Gwenaelle Pound-Lana[b], Matheus Marques Milagre[b,c], Dênia Antunes Saúde-Guimarães[c], José Mário Carneiro Vilela[d], Margareth Spangler Andrade[d], Marta de Lana[a,c], Vanessa Carla Furtado Mosqueira[a,b,c]*

We developed a standardized, precise, selective and accurate method to quantify LYC in pharmaceutical nanoparticulate dosage form and in plasma using a fast and simple HPLC-UV bioanalytical method. This method was successfully applied to the pharmacokinetic analysis of LYC after intravenous administration in mice of different nanocapsules formulations, namely LYC-PCL NC and LYC-PLA-PEG NC, and free LYC. The validation procedure was based on guidelines from FDA for bioanalytical methods *(US Food and Drug Administration GfI, Bioanalytical Method Validation, Centre for Drug Evaluation and Research (CDER) R, 2001).*

Development and validation of the bioanalytical method to quantify lychnopholide (LYC) by HPLC-UV

The method was sensitive to determine the plasmatic concentration of LYC even in small blood volume samples collected from mice. The retention time observed for LYC (3.0 min) and the internal standard itraconazole (IS, 4.5 min) allows a rapid determination of the LYC concentration, which is important for routine analysis, within 6.0 min. Under the proposed chromatographic conditions, no interference was observed between the excipients of nanocapsules (polymers, surfactants and oils) and plasma components as shown in chromatograms (Fig. SI 1). There was no significant interference from endogenous substances at the retention times for LYC and the IS. The chromatographic signals with suitable resolutions are shown in Fig. SI 1**.** The selected IS was detected under the same conditions as LYC owing to its absorption in the UV region (267 nm). The mobile phase contained minimal organic solvent with short analysis times, adequate recoveries and no interference from the plasma matrix. The assays were linear between 0.5 µg/mL and 64 µg/mL in plasma. The mean regression equation of six standard curves was: y = (0.0509) x + (0.0036) for the plasma where y is the peak area ratio of LYC/IS and x is the concentration of LYC (µg/mL). The calibration curves generated showed a good relationship between the area of the peak of LYC/IS (y) and the respective LYC concentration (x), with $r^2 = 0.9998$. The LOQ and LOD were 0.1 µg/mL and 0.05 µg/mL, respectively.

LYC stability in plasma samples

The stability of LYC was evaluated under several conditions: (1) freeze–thaw stability of LYC in plasma through three freeze–thaw cycles; (2) short-term stability of LYC plasma at room temperature for 4 h; and (3) post-preparative stability of LYC during 24 h storage into auto-sampler. All stability tests were performed at the low (1.0 µg/mL), medium (5.0 µg/mL) and high (7.5 µg/mL) QC levels. To evaluate the method sensitivity, LYC lowest limit of quantification (LOQ) and limit of detection (LOD) were also determined.

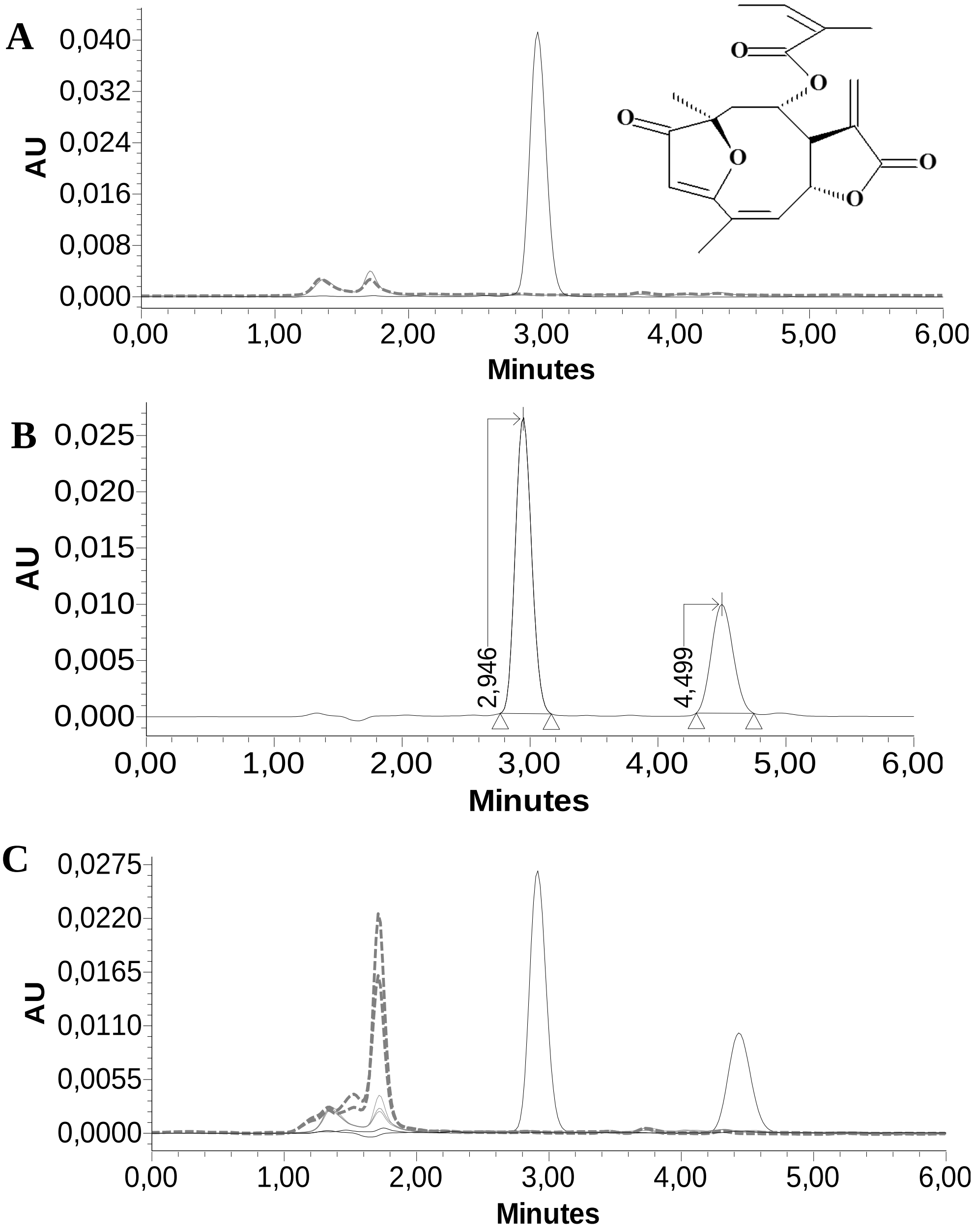

Figure SI 1: Chromatograms of lychnopholide (LYC) loaded in nanocapsules of PLA-PEG (full grey lines) and PCL (dashed grey lines) at 25 µg/mL LYC (**A**). Chromatogram showing the retention times

of LYC and internal standard (itraconazole) at 10.0 µg/mL and 2.5 µg/mL, respectively (**B**). Overlay of the chromatograms of blank plasma sample from healthy mice (full grey line) and plasma (dashed grey lines) spiked with 10 µg/mL of LYC loaded in PLA-PEG NC and 2.5 µg/mL of IS **(C)**.

Table SI 1: Mean recoveries for lychnopholide extraction from quality control plasma samples spiked with lychnopholide (LYC) and internal standard (IS) (n=5)

	Nominal concentration (µg/mL)	Recovery (%)	
	LYC	LYC (CV %)	IS (CV %)
LQC	1.0	110.94 (5.89)	97.62 (3.62)
MQC	5.0	100.65 (3.80)	100.26 (4.26)
HQC	7.5	97.67 (6.96)	97.60 (1.80)
	Mean (µg/mL)	103.08	98.49
	SD (µg/mL)	6.96	1.53
	CV (%)	6.75	1.55

LQC: control quality at the low concentration. MCQ: control quality at the medium concentration. HCQ: control quality at the high concentration. SD: standard deviation. CV: coefficient of variation.

The protein precipitation (PP) method using ACN was chosen because it provided very clean chromatograms and high recovery percentages (99.8-103.8%). Consequently, the PP method was used for LYC and IS extraction from samples. ACN (1 mL) vortex-mixed with plasma sample during 2 min was the best procedure, owing to its low background noise, easiness of sample preparation, and relatively high extraction recovery for analyte and the IS (Table SI 1). The recovery was also very efficient for the IS using the protein precipitation extraction procedure. The mean percentages recovered were 103.08 % for LYC and 98.49 % for the IS in plasma samples. For plasma samples, intra-day precision and accuracy ranged from 1.71 to 5.84 % and 93.10% to 102.06%, respectively. Inter-day precision and accuracy ranged from 2.57 % to 6.45 % and 95.88 % to 99.42 % respectively (Table SI 2). The assay values on both intra- and inter-day were found to be within the acceptance criteria.

Table SI 2: Intra- and inter-day precision and accuracy of the bioanalytical method (HPLC-UV) for the quantification of LYC in mice plasma at three concentration levels (n = 5)

Concentration (µg/mL)		Precision (RSD%)		Accuracy (%)	
		Intra-day	Inter-day	Intra-day	Inter-day
LQC	1.0	1.71	2.57	93.10	97.44
MQC	5.0	5.84	6.45	92.09	95.88
HQC	7.5	2.17	4.45	102.06	99.42

LQC: control quality at the low concentration. MCQ: control quality at the medium concentration. HCQ: control quality at the high concentration. RSD: relative standard deviation. RSD: relative standard deviation. [a]*n*=5. [b]*n*=15

The standard LYC and IS solutions used for the daily preparation of the calibration curve showed no significant degradation during the validation assays. **Table SI 3** lists data for stability tests. The results of the long-term stability analyses (plasma samples stored at -80 °C for 60 days) were evaluated based on the lag time between collecting the plasma samples and the day of analysis. After this period, the samples deviated less than 15% from the nominal concentration, which confirms their stability after extraction. The post-preparative short-term stability (evaluated by keeping the samples at room temperature for 48 h after extraction) and the bench-top short-term stability (LYC extracted from plasma samples kept at room temperature for 4 h) did not indicate significant LYC degradation relative to freshly prepared after extraction. Samples subjected to successive freeze-thaw cycling demonstrated their stability for up to three cycles. All stability determinations were performed using the low, medium and high concentration quality control samples, and the obtained data are shown in **Table SI 3**.

Table SI 3: Stability assays for LYC in mice plasma samples (n=5)

Stability assay	Nominal concentration (µg/mL)	Measured Concentration (µg/mL)	Precision (RSD %)	Accuracy (%)
Long-term (-80°C/60 days)	LQC – 1.0	0.94	1.56	94.30
	MQC – 5.0	4.87	6.02	92.67
	HQC – 7.5	7.39	4.68	97.56
Post-preparative (48 h)	LQC – 1.0	0.96	5.71	101.57
	MQC – 5.0	5.07	4.37	103.35
	HQC – 7.5	7.16	13.47	105.41
Bent-top (4 h)	LQC – 1.0	1.02	8.24	95.42
	MQC – 5.0	4.98	3.84	105.40
	HQC – 7.5	7.47	8.29	99.05
3 Freeze-thaw cycles	LQC – 1.0	0.99	13.88	98.74
	MQC – 5.0	5.19	8.40	107.83
	HQC – 7.5	5.49	5.16	100.12

LQC: control quality at the low concentration. MCQ: control quality at the medium concentration. HCQ: control quality at the high concentration. RSD: relative standard deviation.

Stability of LYC in PBS with 40% mouse plasma at 37°C

Figure SI 2 shows the chromatograms of samples obtained during the LYC release study as described in the manuscript main text.

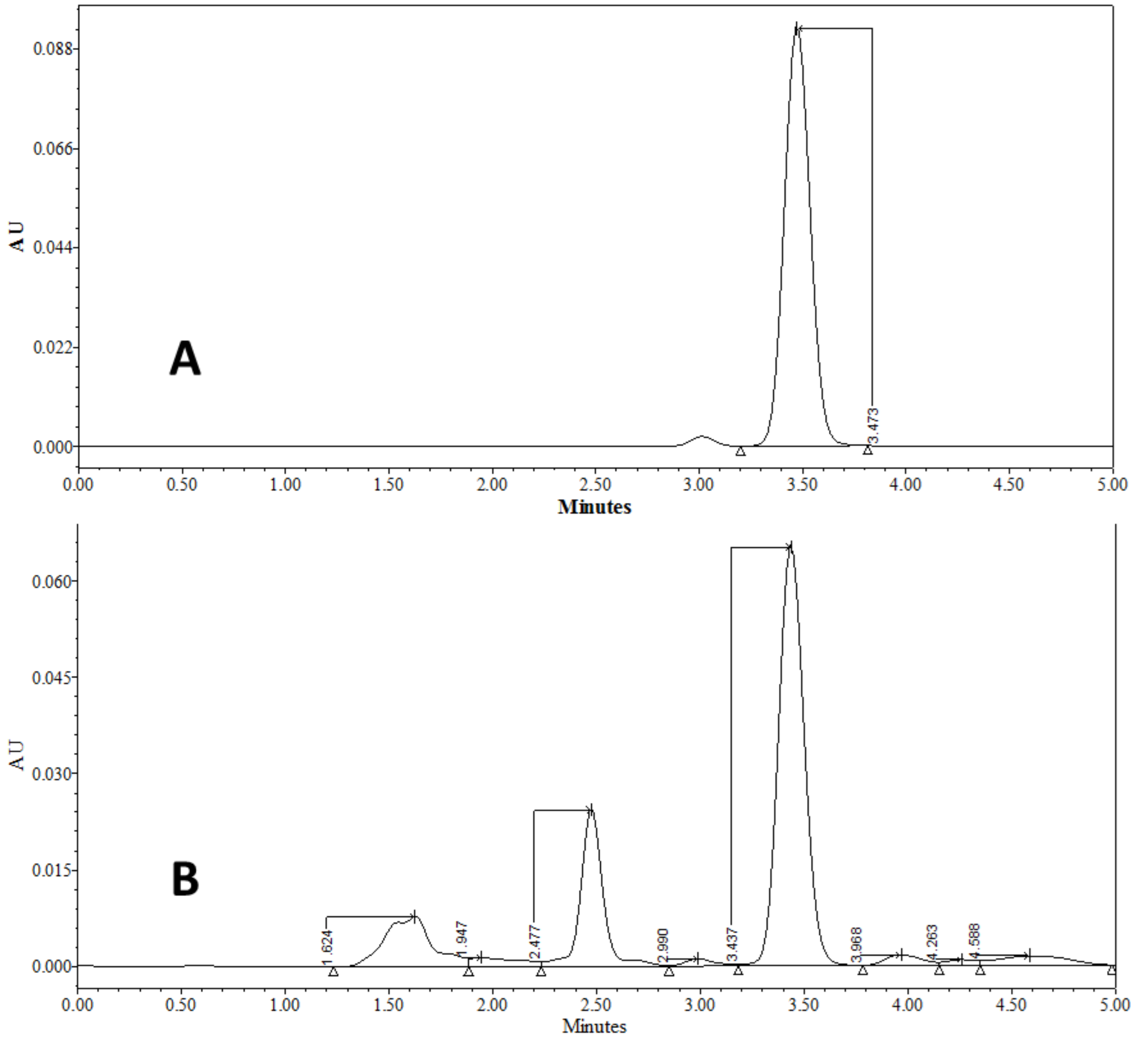

Figure SI 2: Chromatograms of lychnopholide before plasma incubation (**A**) and 24 h after incubation at 37ºC in release medium (PBS pH 7.2) with 40% of mouse plasma (**B**).

Methods

Analyses of calibration standards were conducted in triplicate to assess the linearity. The working solutions were prepared at concentrations of 0.5, 0.75, 1.0, 2.0, 2.5, 5.0, 7.5, 10.0, 16.0, 32.0, 64.0 µg/mL. The specificity of the bioanalytical method was evaluated by comparing the chromatograms of blank mouse plasma (six different batches) to ensure no interference from biological samples at the retention time for IS and LYC, respectively. The calibration curves were generated from the LYC to IS peak area ratios, and linearity was determined by least-squares linear regression over the concentration range of 0.5-10 µg/mL.

Validation parameters

The recovery was calculated for the analyte and for the IS comparing the responses of the analyte from quality control (QC) samples at the concentration of 1.0, 5.0 and 7.5 µg/mL, with the responses of analyte spiked in post-extracted blank plasma at equivalent concentrations. Precision and accuracy were assessed for five different concentrations or quality control (QC), namely, low QC, medium QC and high QC, where the chosen levels were 1.0, 5.0, 7.5 µg/mL, respectively. Intra-run precision and accuracy were calculated for five replicates on the same day and inter-run precision and accuracy for 15 replicate determinations at each QC level, analysed over three non-consecutive days. The QC plasma samples were prepared daily. The analysis of the biological samples and quality control samples were performed within 12-24 h after sample collection.

Lychnopholide extraction from plasma samples

To extract lychnopholide the PP procedure was applied to plasma samples. ACN (900 µL) was added to 90 µL plasma aliquots which were spiked with 10 µL of IS solution. The pool was placed into microcentrifuge tubes, vortexed for 2 min and centrifuged at 9,300 × g for 10 min and the resulting upper organic layers were collected, filtered with PTFE syringe filters of 0.45 µm (Millipore, USA) placed in vials (Waters) and evaporated to dryness in a nitrogen-stream apparatus (TE-019 Concentrator/Tecnal, Brazil). After the evaporation procedure, each sample was reconstituted in 100 µL of the mobile phase and injected (25 µL) in the HPLC system. The recovery was calculated for the analyte and for the IS comparing the responses of the analyte from quality control (QC) samples at the concentration of 1.0, 5.0 and 7.5 µg/mL, with the responses of analyte spiked in post-extracted blank plasma at equivalent concentrations. Precision and accuracy were assessed for five different concentrations or quality control (QC), namely, low QC, medium QC and high QC, where the chosen levels were 1.0, 5.0, 7.5 µg/mL, respectively. Intra-run precision and accuracy were calculated for five replicates on the same day and inter-run precision and accuracy for 15 replicate determinations at each QC level, analysed over three non-consecutive days. The QC plasma samples were prepared daily. The analysis of the biological samples and quality control samples were performed within 12-24 h after sample collection.
